# Endurance exercise training and high-fat diet differentially affect composition of diacylglycerol molecular species in rat skeletal muscle

**DOI:** 10.1152/ajpregu.00371.2017

**Published:** 2018-02-14

**Authors:** Noriaki Kawanishi, Kana Takagi, Hyeon-Cheol Lee, Daiki Nakano, Toshiaki Okuno, Takehiko Yokomizo, Shuichi Machida

**Affiliations:** ^1^Graduate School of Health and Sports Science, Juntendo University, Inzai, Chiba, Japan; ^2^Research Fellow of the Japan Society for the Promotion of Sciences, Tokyo, Japan; ^3^Institute of Health and Sports Science and Medicine, Juntendo University, Inzai, Japan; ^4^Faculty of Advanced Engineering, Chiba Institute of Technology, Narashino, Chiba, Japan; ^5^Department of Biochemistry, Graduate School of Medicine, Juntendo University, Tokyo, Japan

**Keywords:** athlete’s paradox, diacylglycerol, skeletal muscle

## Abstract

Insulin resistance of peripheral muscle is implicated in the etiology of metabolic syndrome in obesity. Although accumulation of glycerolipids, such as triacylglycerol and diacylglycerol (DAG), in muscle contributes to insulin resistance in obese individuals, endurance-trained athletes also have higher glycerolipid levels but normal insulin sensitivity. We hypothesized that the difference in insulin sensitivity of skeletal muscle between athletes and obese individuals stems from changes in fatty acid composition of accumulated lipids. Here, we evaluated the effects of intense endurance exercise and high-fat diet (HFD) on the accumulation and composition of lipid molecular species in rat skeletal muscle using a lipidomic approach. Sprague-Dawley female rats were randomly assigned to three groups and received either normal diet (ND) in sedentary conditions, ND plus endurance exercise training, or HFD in sedentary conditions. Rats were fed ND or HFD between 4 and 12 wk of age. Rats in the exercise group ran on a treadmill for 120 min/day, 5 days/wk, for 8 wk. Soleus muscle lipidomic profiles were obtained using liquid chromatography/tandem mass spectrometry. Total DAG levels, particularly those of palmitoleate-containing species, were increased in muscle by exercise training. However, whereas the total DAG level in the muscle was also increased by HFD, the levels of DAG molecular species containing palmitoleate were decreased by HFD. The concentration of phosphatidylethanolamine molecular species containing palmitoleate was increased by exercise but decreased by HFD. Our results indicate that although DAG accumulation was similar levels in trained and sedentary obese rats, specific changes in molecular species containing palmitoleate were opposite.

## INTRODUCTION

Obesity is a major risk factor for metabolic syndromes such as dyslipidemia, hypertension, arteriosclerosis, and type 2 diabetes, which are highly prevalent worldwide. Insulin resistance has been implicated in the etiology of metabolic syndrome in obesity. Although the mechanisms of insulin resistance in peripheral tissues, such as the muscle and liver, are still not fully understood, ectopic lipid accumulation, including an increase in intracellular lipids, has been proposed as a possible explanation. Recently, increased concentrations of intramyocellular lipids have been demonstrated in sedentary obese individuals by proton magnetic resonance spectroscopy (^1^H-MRS) and muscle biopsy (12, 24, 28). Importantly, the level of intramyocellular lipids negatively correlated with insulin sensitivity in obese individuals (11, 23). Therefore, intramyocellular lipid accumulation may be considered as a direct or indirect cause of insulin resistance. For these reasons, lipid levels in skeletal muscle are now used as biomarkers of diseases such as diabetes.

Myocellular lipids include diacylglycerol (DAG), triacylglycerol (TAG), and phospholipids. Interestingly, obese humans and rodents display higher levels of DAG in skeletal muscle than normal individuals (18, 22, 34). DAG has been shown to induce dysfunction of insulin signaling by suppressing phosphorylation of insulin receptor substrate in muscle cells (32). Moreover, Chibalin et al. (7) showed that in mice deficient in diacylglycerol kinase-δ, a key attenuator of DAG level, insulin-stimulated glucose uptake in skeletal muscle was inhibited.

Glycerolipids such as TAG and DAG are composed of glycerol and fatty acids. The molecular identity of the latter component determines the nature of glycerolipid effects on skeletal muscle. For example, mice infused with TAG containing palmitate (16:0) moiety showed impaired insulin signaling in skeletal muscle (6). Conversely, infusion with TAG containing palmitoleate (16:1) activated the insulin-signaling pathway in skeletal muscle (6). The results of that study strongly suggest that skeletal muscle insulin resistance is affected by the alteration of fatty acid composition of glycerolipids. Importantly, it has been recently shown that the content of DAG containing palmitate in skeletal muscle was elevated after high-fat diet (HFD) consumption (21). Therefore, accumulation of DAG containing palmitate in skeletal muscle may play an important role in obesity-induced insulin resistance.

Although those results indicate that DAG accumulation in skeletal muscle contributes to insulin resistance, recent studies also revealed higher DAG levels in skeletal muscle of endurance-trained athletes (1). However, insulin sensitivity of endurance-trained athletes was not impaired (1). This phenomenon has been known as the “athlete’s paradox.” Previous studies have shown that endurance exercise training markedly increases skeletal muscle lipid content in humans and rodents. For example, in healthy lean subjects, lipid content in skeletal muscle increases after endurance exercise training (3, 15, 33). Moreover, endurance exercise training has been shown to increase DAG content in rat skeletal muscle (9). Those studies strongly suggest that endurance exercise training promotes lipid accumulation in skeletal muscle. We hypothesized that the difference in skeletal muscle insulin sensitivity, despite similar levels of DAG accumulation, in athletes and obese individuals might be associated with dissimilar lipid accumulation and alterations of fatty acid composition of glycerolipids. Endurance exercise training is known to increase total glycerolipid content in skeletal muscle. However, the effect of endurance exercise training on fatty acid composition of glycerolipids has not been yet evaluated. It has been reported that 4 wk of moderate intensity endurance exercise training did not alter fatty acid composition of TAG and DAG in mouse skeletal muscle (21). However, unlike in other previous studies, total skeletal muscle DAG content was not affected by training in that model. Here, we used lipidomic approach to explore potential differences between the effects of high-intensity endurance exercise training and HFD consumption on total lipid content and composition of lipid molecular species in rat skeletal muscle.

## METHODS

### 

#### Animals.

Sprague-Dawley female rats (*n* = 48) were purchased from SLC Japan at 3 wk of age and housed in groups of two rats per cage in a controlled environment under reverse light-dark cycle (lights on at 2100 and off at 900). The experimental procedures complied with the *Guiding Principles for the Care and Use of Animals* of the Juntendo University and were approved by the Institutional Animal Care and Use Committee of the University (No. H27-3). The rats were randomly assigned to three groups (*n* = 16/each groups) that received either normal diet plus sedentary (ND/Sed) group, ND plus endurance exercise-training (ND/Ex) group, or HFD plus sedentary (HFD/Sed) group. HFD caloric composition comprised 60% calories from fats, 20% from proteins, and 20% from carbohydrates (D12492; Research Diets, New Brunswick, NJ). The rats were fed HFD from 4 to 12 wk of age. ND rats were fed standard chow of the following caloric composition: 10% fat, 20% protein, and 70% carbohydrates (D12450B; Research Diets). The contents of free fatty acid are shown in Table 1. The contents of some long-chain fatty acids [e.g., myristate (C14:0), palmitate (C16:0), palmitoleate (C16:1), stearate (C18:0), oleate (C18:1), and linoleate (C18:2)] of HFD are 4- to 10-fold higher than those of ND (Table 1). All groups had free access to food and water. The animals were weighed weekly, and food intake per each cage with two rats was measured monthly.

**Table 1.** Fatty acid content of normal diet and high-fat diet

	ND, mg/g	HFD, mg/g
C10:0	0	0.1
C12:0	0	0.2
C14:0	0.2	2.8
C15:0	0	0.2
C16:0	6.5	49.9
C16:1	0.3	3.4
C17:0	0.1	0.9
C18:0	3.1	26.9
C18:1	12.6	86.6
C18:2	18.3	73.1
C18:3	2.2	5.2
C20:0	0	0.4
C20:1	0.1	1.5
C20:2	0.2	2
C20:3	0	0.3
C20:4	0.1	0.7
C22:5	0	0.2
Total	43.7	254.5

ND, normal diet; HFD, high-fat diet.

Endurance exercise training was initiated when the rats were 4 wk of age and continued until 12 wk of age. Before the experiment, the rats performed treadmill running for 15 min once during the acclimatization period. For exercise training, rats were placed on a motorized treadmill (Natsume, Kyoto, Japan) for 30–120 min/day (during the dark phase) 5 days/wk. The exercise speed during the first 4 wk was set at 21–31 m/min and during the remaining 4 wk at 31 m/min.

#### Maximal oxygen uptake measurements.

Rats of three group (*n* = 8/each groups) were treadmill tested to measure indexes defining exercise capacity as previously described. At the time of treadmill testing, each rat was placed on a treadmill enclosed by a metabolic chamber, through which air flowing at a constant speed (2 l/min) was passed (ARCO-2000). Oxygen and carbon dioxide gas fractions were monitored at both the inlet and output ports of the metabolic chamber. Basal measurements were obtained over a period of 10 min. After basal measurements, the angle was fixed at 8.5°, and the speed was increased incrementally by 5 m/min every 2 min until the rats reached exhaustion. Exhaustion was defined as the point when the mouse refused to run despite being given the shock grid five times. Whole body oxygen uptake was automatically calculated every 60 s.

#### Muscle sample preparation for mass spectrometry, PCR, and Western blot.

Twelve-week-old rats of three groups (*n* = 8/groups) were killed in 2 days after the final exercise-training session under light isoflurane anesthesia. All rats were killed after 3 h of being fasted. The skeletal muscle and adipose tissues were quickly removed, weighed, frozen in liquid nitrogen, and stored at –80°C until analysis.

#### Mass spectrometry analysis.

Tissues (30–60 mg) were crushed to powder with an SK mill (SK-100; Tokken, Chiba, Japan) without thawing, and lipids were extracted by the method of Bligh and Dyer (5) with internal standards. The organic (lower) phase was transferred to a clean vial and dried under a stream of nitrogen. The lipids were then resolubilized in 200 µl of 2:1 (vol/vol) CHCl_3_/MeOH and stored at −80°C. Before lipid analysis, 10 µl of lipid samples were further diluted with 40 µl of acetonitrile, and 2 µl were injected onto the liquid chromatography/tandem mass spectrometry (LC-MS/MS) system. LC separation was performed on an ACQUITY UPLC BEH C_18_ column (1.7 µm, 2.1 × 100 mm; Waters, Milford, MA) coupled to an ACQUITY UPLC BEH C_18_ VanGuard Pre-column (1.7 µm, 2.1 × 5 mm; Waters). Mobile *phase A* was 60:40 (vol/vol) acetonitrile/H_2_O with 10 mM ammonium formate and 0.1% (vol/vol) formic acid, and mobile *phase B* was 90:10 (vol/vol) isopropanol/acetonitrile with 10 mM ammonium formate and 0.1% (vol/vol) formic acid. LC gradient consisted of 20% *B* for 2 min, a linear gradient to 60% *B* over 4 min, a linear gradient to 100% *B* over 16 min, and equilibration with 20% *B* for 3 min (25 min total run time). The flow rate was 0.3 ml/min and column temperature was 55°C. Multiple reaction monitoring (MRM) was performed using a Xevo TQ-S micro triple quadrupole mass spectrometry system (Waters) equipped with an electrospray ionization source. Electrospray ionization capillary voltage was set at 1.0 kV, and the sampling cone was set at 30 V. The source temperature was set at 150°C, desolvation temperature was set at 500°C, and desolvation gas flow was 1,000 l/h. The cone gas flow was set at 50 l/h. For DAGs and TAGs, MRM transition from [M+NH_4_]^+^ to the fragment ion [M-RCOOH-NH_3_]^+^ (RCOOH, fatty acid) with collision energy 17 V (DAGs) or 25 V (TAGs) was used. Absolute abundances of DAGs and TAGs were estimated by comparison to that of 17:1/17:1 DAG (diheptadecenoin; Nu-Chek Prep, Elysian, MN) or 17:1/17:1/17:1 TAG (triheptadecenoin; Nu-Chek Prep) standard, respectively. For phosphatidylcholine (PC) and phosphoethanolamine (PE), MRM transition from [M+HCOO]^−^ (PC) or [M-H]^−^ (PE) to the fragment ion [RCOO]^−^ with collision energy 38 V (PC) or 30 V (PE) was used. Relative abundances of PC and absolute abundances of PE were estimated by comparison to 15:0/15:0 PE (1,2-dipentadecanoyl-*sn*-glycero-3-phosphoethanolamine; Avanti Polar Lipids, Alabaster, AL), respectively. The relative value of the total levels of each lipid class was calculated based on the sum value of all the molecular species concentrations detected via mass spectrometry.

#### Real-time quantitative PCR.

Total RNA was extracted from soleus muscle homogenate using an RNeasy Fibrosis Mini Kit (Qiagen, Valencia, CA) according to the manufacturer’s instructions. The purity and quantity of total RNA were assessed using a NanoDrop system (NanoDrop Technologies, Wilmington, DE). Total RNA was reverse transcribed into cDNA using a high-capacity cDNA Reverse Transcription Kit (Applied Biosystems, Waltham, MA) according to the manufacturer’s instructions. PCR was performed with a 7300 real-time PCR system (Applied Biosystems) using PowerUp SYBR Green PCR Master Mix (Applied Biosystems). The thermal profiles consisted of denaturation at 95°C for 10 min followed by 40 cycles of 95°C for 15 s and annealing at 60°C for 60 s. The expression of β-actin mRNA was used as a housekeeping control, and all data were normalized to the β-actin mRNA level. Data are expressed as fold change relative to the values of ND/Sed group. Specific PCR primer pairs for each gene are shown in Table 2.

**Table 2.** Primer sequences for real-time PCR analysis

Gene	Forward	Reverse
*β-Actin*	CATGAAGATCAAGATCATTGCTCCT	CTGCTTGCTGATCCACATCTG
*MGAT1*	AAGGAAGGAGGTGGGAACAT	AATCCTTTGCGCTGAAGAAG
*MGAT2*	GGTGAGTGCTGATCACATTCTGA	AAGGGAGATGCCCATGATCT
*MGAT3*	AGCCTGGAACAGTGAGTTTGC	GGCTTCGCTGCACTGGAA
*DGAT1*	TATTACTTCATCTTTGCTCC	AAAGTAGGTGACAGACTCAG
*DGAT2*	TGCCCGCAGCGAGAACAAGAATAAA	GGCGTGTTCCAGTCAAATGCCA
*SCD-1*	CCTTAACCCTGAGATCCCGTAGA	AGCCCATAAAAGATTTCTGCAAA

MGAT, monoacylglycerol acyltransferase; DGAT, diacylglycerol acyltransferase; SCD-1 stearoyl-CoA desaturase 1.

#### Western blot.

Soleus muscle was homogenized (Polytron) in ice-cold RIPA buffer (25 mM Tris·HCl, 150 mM NaCl, 1% NP-40, 1% sodium deoxycholate, and 0.1% SDS, pH 7.6) supplemented with Halt Protease Inhibitor Cocktail (Thermo Fisher Scientific). Homogenates were centrifuged at 14,000 *g* for 15 min, and the supernatant was analyzed. Protein concentration of muscle lysates was subsequently determined using a BCA Protein Assay Kit (Thermo Fisher Scientific). Muscle lysates were solubilized in Laemmli sample buffer (Bio-Rad) and heated to 100°C for 5 min. The samples (20 μg protein) were subjected to 10% SDS-PAGE, transferred to a PVDF membrane, blocked with PVDF blocking reagent (TOYOBO), and incubated with primary antibodies against stearoyl-CoA desaturase 1 (SCD1; 1:200 dilution; Santa Cruz Biotechnology) and α-tubulin (1:2,000 dilution; Abcam) overnight and, finally, with a horseradish peroxide-linked secondary anti-rabbit IgG antibody (1:5,000 dilution; Abcam). Blotted samples were analyzed using EzWestLumi plus (ATTO) and quantified by densitometry.

#### Statistical analysis.

All data are presented as the means ± SE. Statistical analysis was carried out using one-way ANOVA followed by Dunnett’s multiple comparison test. The results obtained were compared with the ND plus sedentary (ND/Sed) rats. Differences were considered significant if *P* < 0.05.

## RESULTS

### 

#### Effects of exercise training and HFD on body mass and fat mass.

Exercise training decreased retroperitoneal and parametrial fat mass but did not affect body mass (Table 3). HFD markedly increased body mass, and this effect was associated with the increase in fat mass of the kidney leaf, as well as retroperitoneal and parametrial fat. Exercise training and HFD significantly increased soleus muscle mass, whereas liver mass was not changed.

**Table 3.** Body mass, muscle mass, calorie intake, muscle mass, fat mass, and liver mass of normal diet/sedentary, normal diet/exercise-training, and high-fat diet/sedentary rats

	ND/Sed	ND/Ex	HFD/Sed
Initial body mass, g	66.4 ± 1.2	66.1 ± 1.6	67.5 ± 1.2
Final body mass, g	254.7 ± 6.5	249.5 ± 9.2	313.5 ± 11.6*
Calorie intake, kcal/wk	414.7 ± 8.9	418.9 ± 8.5	553.3 ± 17.2*
Soleus muscle mass, mg	109.2 ± 2.4	124.5 ± 4.7*	122.5 ± 2.4*
Soleus muscle mass/body mass	0.43 ± 0.01	0.48 ± 0.01*	0.39 ± 0.02
Kidney leaf fat mass, g	0.98 ± 0.07	0.68 ± 0.05*	1.76 ± 0.20*
Retroperitoneal fat mass, g	1.96 ± 0.08	1.30 ± 0.08*	3.65 ± 0.26*
Parametrial fat mass, g	11.08 ± 0.55	7.06 ± 0.77*	17.01 ± 1.52*
Kidney leaf fat mass/body mass	3.81 ± 0.43	2.75 ± 0.17*	5.56 ± 0.83
Retroperitoneal fat mass/body mass	7.63 ± 0.30	5.29 ± 0.46*	11.53 ± 0.82*
Parametrial fat mass/body mass	43.48 ± 1.79	28.49 ± 3.13*	53.78 ± 3.40*
Liver mass, g	0.93 ± 0.06	0.93 ± 0.05	0.93 ± 0.07

Data are presented as means ± SE. ND/Sed, normal diet/sedentary rats; ND/Ex, normal diet/exercise-training rats; HFD/Sed, high-fat diet/sedentary rats.

* *P* < 0.05 compared with the ND/Sed rats by Dunnett's multiple comparison test.

#### Effects of exercise training and HFD on maximal oxygen uptake and peroxisome proliferator-activated receptor-γ coactivator expression levels.

**Fig. 1.**
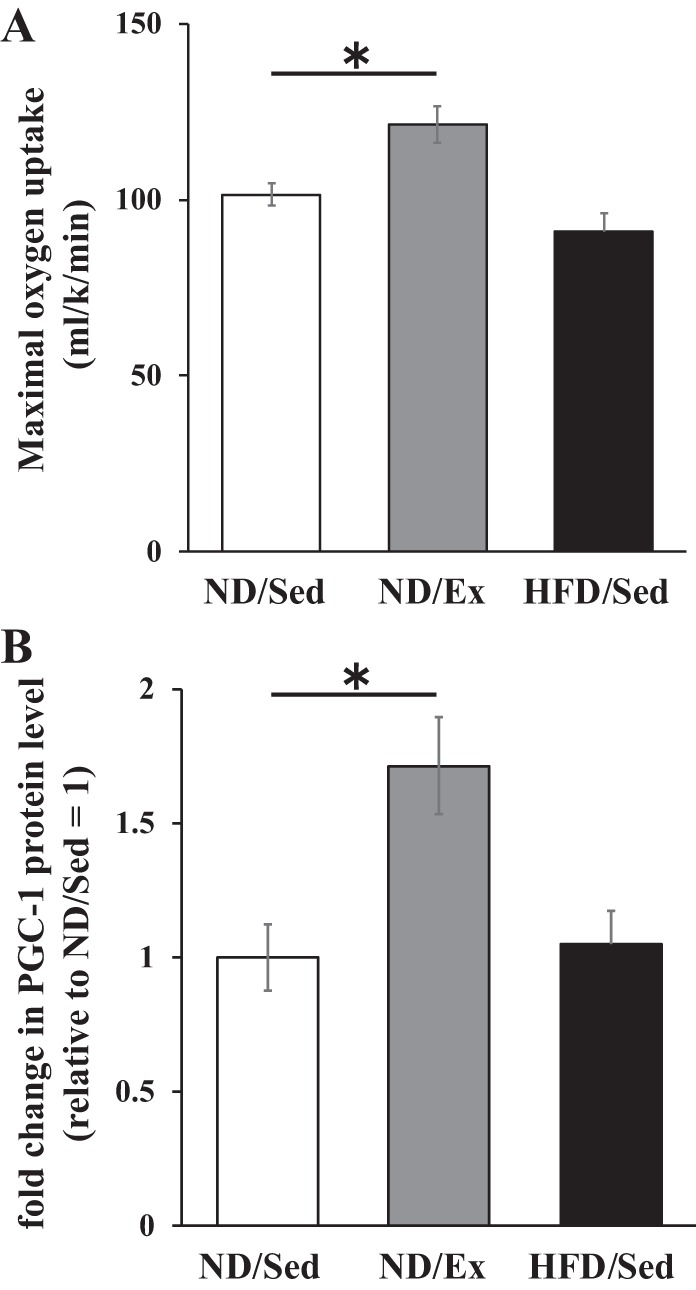
Effects of endurance exercise training and high-fat diet (HFD) on maximal oxygen uptake and peroxisome proliferator-activated receptor-γ coactivator (PGC-1) protein expression in skeletal muscle. *A*: maximal oxygen uptake of normal diet (ND) plus sedentary (ND/Sed) rats, ND plus endurance exercise-training (ND/Ex) rats, and HFD plus sedentary (HFD/Sed) rats. *B*: relative protein levels of PGC-1 in soleus muscle of ND/Sed rats, ND/Ex rats, and HFD/Sed rats. Data are presented as means ± SE. *P < 0.05 compared with the ND/Sed rats by Dunnett’s multiple comparison test.

We evaluated exercise capacity by maximal oxygen uptake measurements. We observed that maximal oxygen uptake level was higher in ND-fed exercise-training rats than in ND sedentary rats (Fig. 1*A*), whereas these levels were similar in sedentary rats that received ND and HFD (Fig. 1*A*). In this study, we also found that peroxisome proliferator-activated receptor-γ coactivator (PGC-1) expression levels in skeletal muscle level was markedly increased by exercise training (Fig. 1*B*), whereas these expression levels were not affected by HFD (Fig. 1*B*).

#### Effects of exercise training and HFD on lipid concentration and composition of DAG lipid molecular species in skeletal muscle.

**Fig. 2.**
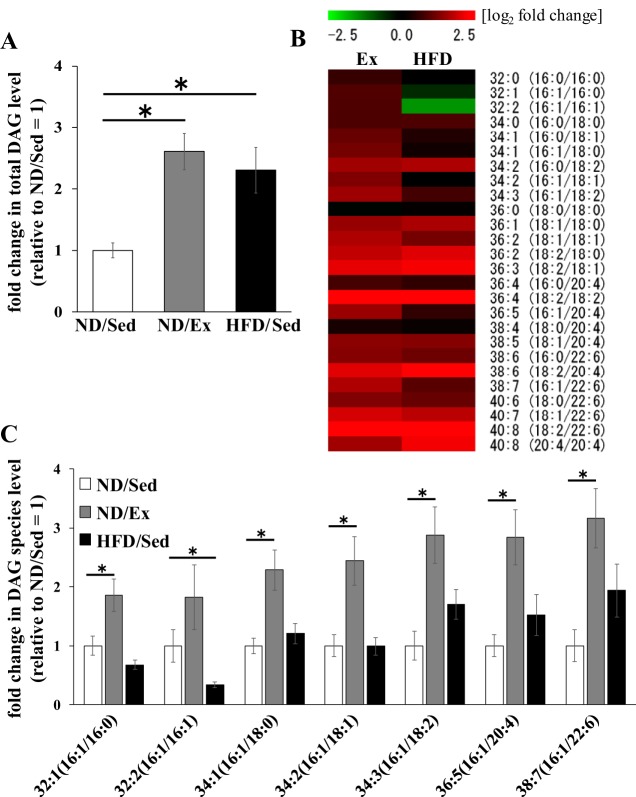
Effects of endurance exercise training and high-fat diet (HFD) on diacylglyceride (DAG) content in skeletal muscle. *A*: total DAG content in soleus muscle of normal (ND) plus sedentary (ND/Sed) rats, ND plus endurance exercise-training (ND/Ex) rats, and HFD plus sedentary (HFD/Sed) rats. *B*: heat map showing the fold change of DAG molecular species content in soleus muscle of ND/Ex and HFD/Sed rats in comparison to the corresponding values in ND/Sed rats. *C*: relative levels of DAG molecular species in soleus muscle of ND/Sed rats, ND/Ex rats, and HFD/Sed rats. Data are presented as mean ± SE. *P < 0.05 compared with the ND/Sed rats by Dunnett’s multiple comparison test.

We evaluated lipid molecular species in skeletal muscle by LC-MS/MS. We identified 11 lipid classes (TAG, DAG, PE, PC, lysophosphatidylethanolamine, lysophosphatidylcholine, ceramide, sphingomyelin, acylcarnitine, coenzyme Q, and cholesterylester) by lipidomic analysis. Importantly, we found differences in glycerolipid and phospholipid concentrations in the identified lipid classes. We have also identified detailed molecular species of each lipid by LC-MS/MS. Concentrations of different molecular species of DAG in muscle tissue of the three groups of rats are shown in Table 4. We observed that total DAG concentrations in the muscle of exercise-trained rats and rats on HFD were higher than that in the muscle of untrained rats on ND (Fig. 2*A*). However, as illustrated by the heat map, the variation patterns of DAG molecular species were different (Fig. 2*B*). Relative levels of DAG molecular species containing C16:1 (palmitoleate) in rats that underwent exercise training or received HFD are shown in Fig. 2*C*. Importantly, exercise training was associated with higher relative concentrations of DAG molecular species containing C16:1 (e.g., DAG 32:1, 34:2, 34:2, 34:3, 36:5, and 38:6) than those in ND-fed sedentary rats (Fig. 2*C*). In contrast to the pattern observed in exercise-trained muscle, the relative concentrations of DAG molecular species containing C16:1 (DAG 32:2) in HFD rats were lower than those in ND sedentary animals (Fig. 2*C*).

**Table 4.** Diacylglycerol molecular species levels in soleus muscle of normal diet/sedentary, normal diet/exercise-training, and high-fat diet/sedentary rats

	*m*/*z*					
Species	Precusor ion	Product ion	Adduct Ions	ND/Sed, nmol/g tissue	ND/Ex, nmol/g tissue	HFD/Sed, nmol/g tissue
32:0 (16:0/16:0)	586.54	313.27	[M+NH_4_]^+^	3.70 ± 0.36	5.81 ± 0.53*	3.68 ± 0.39
32:1 (16:1/16:0)	584.53	313.27	[M+NH_4_]^+^	3.11 ± 0.51	5.77 ± 0.87*	2.09 ± 0.24
32:2 (16:1/16:1)	582.51	311.26	[M+NH_4_]^+^	0.344 ± 0.095	0.627 ± 0.191	0.115 ± 0.015*
34:0 (16:0/18:0)	614.57	341.31	[M+NH_4_]^+^	1.25 ± 0.05	2.27 ± 0.19*	2.27 ± 0.21*
34:1 (16:0/18:1)	612.56	339.29	[M+NH_4_]^+^	30.7 ± 3.4	64.6 ± 6.5*	41.8 ± 4.9
34:1 (16:1/18:0)	612.56	341.31	[M+NH_4_]^+^	0.417 ± 0.055	0.954 ± 0.142*	0.504 ± 0.072
34:2 (16:0/18:2)	610.54	337.27	[M+NH_4_]^+^	14.9 ± 2.7	43.8 ± 5.6*	46.4 ± 7.8*
34:2 (16:1/18:1)	610.54	339.29	[M+NH_4_]^+^	7.3 ± 1.4	17.8 ± 3.0*	7.2 ± 1.1
34:3 (16:1/18:2)	608.53	337.27	[M+NH_4_]^+^	0.80 ± 0.20	2.30 ± 0.38*	1.36 ± 0.20
36:0 (18:0/18:0)	642.60	341.31	[M+NH_4_]^+^	0.284 ± 0.037	0.271 ± 0.018	0.301 ± 0.024
36:1 (18:1/18:0)	640.59	341.31	[M+NH_4_]^+^	3.69 ± 0.36	10.20 ± 1.11*	11.38 ± 1.69*
36:2 (18:1/18:1)	638.57	339.29	[M+NH_4_]^+^	28.5 ± 4.3	89.8 ± 11.4*	65.0 ± 11.3*
36:2 (18:2/18:0)	638.57	341.31	[M+NH_4_]^+^	8.2 ± 0.7	28.5 ± 3.6*	33.2 ± 5.2*
36:3 (18:2/18:1)	636.56	339.29	[M+NH_4_]^+^	21.7 ± 3.8	91.7 ± 12.9*	98.8 ± 20.3*
36:4 (16:0/20:4)	634.54	361.27	[M+NH_4_]^+^	3.40 ± 0.28	5.87 ± 0.50*	5.14 ± 0.81
36:4 (18:2/18:2)	634.54	337.27	[M+NH_4_]^+^	1.37 ± 0.25	6.64 ± 0.94*	9.42 ± 2.12*
36:5 (16:1/20:4)	632.53	361.27	[M+NH_4_]^+^	0.130 ± 0.025	0.369 ± 0.061*	0.198 ± 0.046
38:4 (18:0/20:4)	662.57	361.27	[M+NH_4_]^+^	31.7 ± 1.9	40.3 ± 2.5*	36.4 ± 1.7
38:5 (18:1/20:4)	660.56	361.27	[M+NH_4_]^+^	3.20 ± 0.43	8.33 ± 0.99*	8.02 ± 1.65*
38:6 (16:0/22:6)	658.54	385.27	[M+NH_4_]^+^	2.01 ± 0.38	5.05 ± 0.54*	4.40 ± 0.92*
38:6 (18:2/20:4)	658.54	361.27	[M+NH_4_]^+^	0.52 ± 0.11	2.12 ± 0.34*	3.86 ± 0.95*
38:7 (16:1/22:6)	656.53	385.27	[M+NH_4_]^+^	0.246 ± 0.066	0.779 ± 0.123*	0.477 ± 0.110
40:6 (18:0/22:6)	686.57	385.27	[M+NH_4_]^+^	2.40 ± 0.31	5.90 ± 0.59*	5.07 ± 0.79*
40:7 (18:1/22:6)	684.56	385.27	[M+NH_4_]^+^	1.88 ± 0.53	7.24 ± 1.01*	6.32 ± 1.52*
40:8 (18:2/22:6)	682.54	385.27	[M+NH_4_]^+^	0.54 ± 0.15	2.61 ± 0.40*	3.17 ± 0.78*
40:8 (20:4/20:4)	682.54	361.27	[M+NH_4_]^+^	0.221 ± 0.042	0.650 ± 0.107*	0.993 ± 0.246*

Data are presented as means ± SE. ND/Sed, normal diet/sedentary rats; ND/Ex, normal diet/exercise-training rats; HFD/Sed, high-fat diet/sedentary rats.

* *P* < 0.05 compared with the ND/Sed rats by Dunnett's multiple comparison test.

#### Effects of exercise training and HFD on TAG concentration and composition of TAG molecular species in skeletal muscle.

**Fig. 3.**
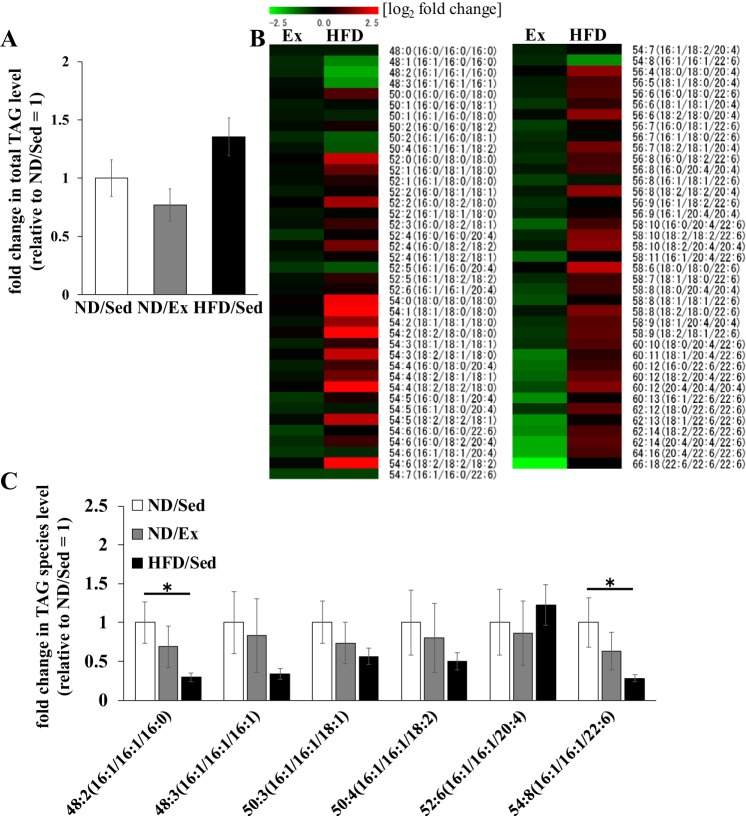
Effects of endurance exercise training and high-fat diet (HFD) on triacylglyceride (TAG) content in skeletal muscle. *A*: total TAG content in soleus muscle of normal diet ND) plus sedentary (ND/Sed) rats, ND plus endurance exercise-training (ND/Ex) rats, and HFD plus sedentary (HFD/Sed) rats. *B*: heat map showing the fold change of TAG molecular species content in soleus muscle of ND/Ex and HFD/Sed rats in comparison to the corresponding values in ND/Sed rats. *C*: relative levels of TAG molecular species in soleus muscle of ND/Sed rats, ND/Ex rats, and HFD/Sed rats. Data are presented as means ± SE. *P < 0.05 compared with the ND/Sed rats by Dunnett’s multiple comparison test.

The total TAG concentration in soleus muscle was similar in sedentary ND-fed rats and ND-fed rats that underwent exercise training (Fig. 3*A*). TAG molecular species composition was not affected by exercise training in ND-fed rats, whereas HFD led to several changes: the levels of TAG molecular species containing C16:0, C18:0, C18:1, and C18:2 were higher in HFD-fed rats than in ND sedentary rats. However, the variation pattern of TAG molecular species containing C16:1 was different (Fig. 3*B*). The relative concentrations of TAG molecular species containing two or three palmitoleate moieties in muscle tissue of ND-fed rats that underwent exercise training and of HFD-fed rats are shown in Fig. 3*C*. Exercise training did not significantly alter the levels of any TAG molecular species containing C16:1 in ND-fed sedentary rats (Fig. 3*C*), whereas concentrations of C16:1 containing TAGs (48:2 and 54:8) were remarkably lower in soleus muscle of HFD-fed rats (Fig. 3*C*).

#### Effects of exercise training and HFD on the composition of PE and PC molecular species in skeletal muscle.

**Fig. 4.**
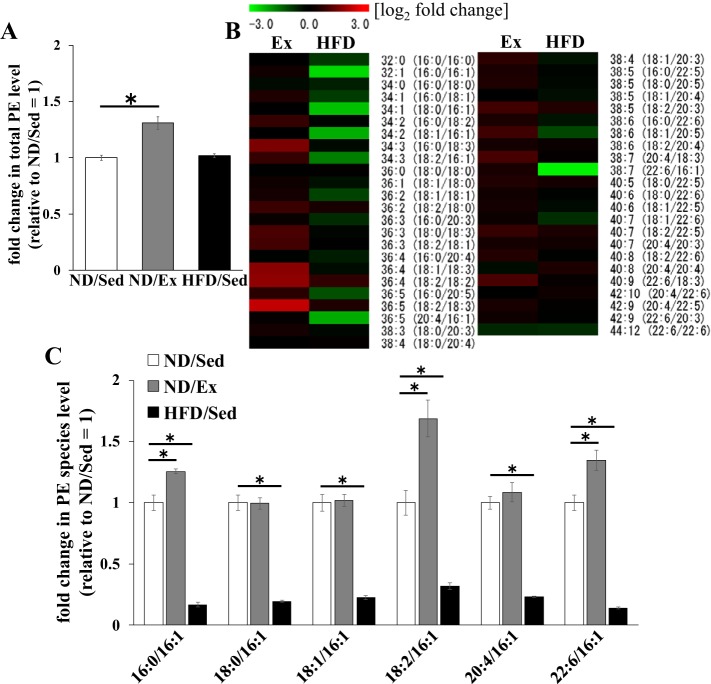
Effects of endurance exercise training and high-fat diet (HFD) on phosphatidylethanolamine (PE) content in skeletal muscle. *A*: total PE content in soleus muscle of normal diet (ND) plus sedentary (ND/Sed) rats, ND plus endurance exercise-training (ND/Ex) rats, and HFD plus sedentary (HFD/Sed) rats. *B*: heat map showing the fold change of PE molecular species content in soleus muscle of ND/Ex and HFD/Sed rats in comparison to the corresponding values in ND/Sed rats. *C*: relative levels of PE molecular species in soleus muscle of ND/Sed rats, ND/Ex rats, and HFD/Sed rats. Data are presented as means ± SE. *P < 0.05 compared with the ND/Sed rats by Dunnett’s multiple comparison test.

**Fig. 5.**
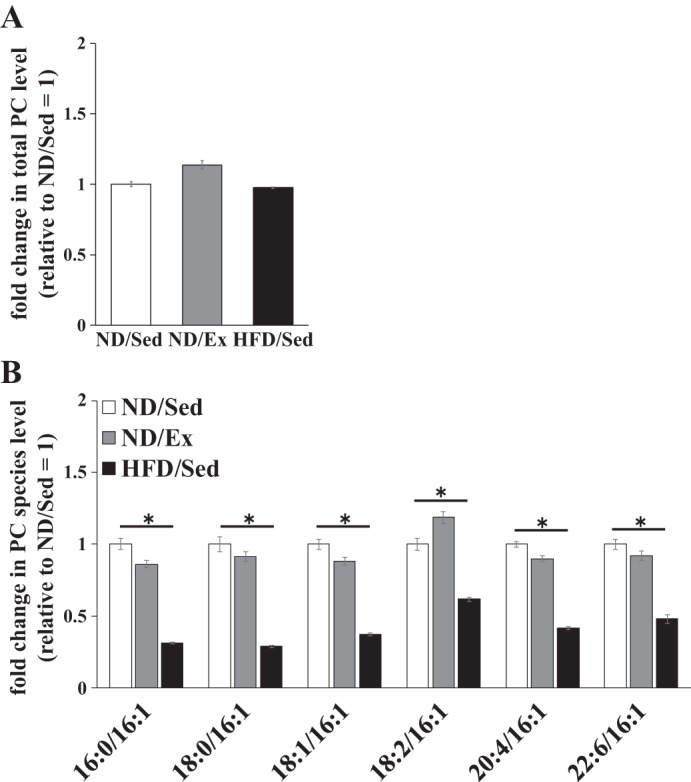
Effects of endurance exercise training and high-fat diet (HFD) on phosphatidylcholine (PC) content in skeletal muscle. *A*: total PC content in soleus muscle of normal diet (ND) plus sedentary (ND/Sed) rats, ND plus endurance exercise training (ND/Ex) rats, and HFD plus sedentary (HFD/Sed) rats. *B*: relative levels of PC molecular species in soleus muscle of ND/Sed rats, ND/Ex rats, and HFD/Sed rats. Data are presented as means ± SE. *P < 0.05 compared with the ND/Sed rats by Dunnett’s multiple comparison test.

Concentration of total PE in soleus muscle of ND-fed rats was increased by exercise training (*P* < 0.01), whereas HFD consumption did not have a significant effect (Fig. 4*A*). As can be seen from the heat map, the relative levels of multiple PE molecular species were different in exercise-trained and untrained muscle of ND-fed rats (Fig. 4*B*). In HFD-fed rats, the relative levels of PE molecular species containing C16:1 were significantly different from those in ND-fed sedentary rats (Fig. 4*B*). ND-fed rats that had exercise training had higher concentrations of PE molecular species containing C16:1 (e.g., PE 32:1, 34:3, and 38:7) in soleus muscle (Fig. 4*C*). In contrast, HFD consumption led to lower levels of PE molecular species containing C16:1 (PE 32:1, 34:1, 34:2, 34:3, 36:5, and 38:7) compared with those in ND-fed sedentary rats (Fig. 4*C*). Total PC concentrations in soleus muscle were not affected by exercise training and HFD (Fig. 5*A*). As in the case of PE molecular species, the relative concentrations of PC species containing C16:1 (PC 32:1, 34:1, 34:2, 34:3, 36:5, and 38:7) in HFD-fed rats were lower than those in ND-fed sedentary rats (Fig. 5*B*). However, the relative concentrations of PC molecular species were similar between sedentary ND-fed rats and ND-fed animals that underwent exercise training (Fig. 5*B*).

#### Effects of exercise training and HFD on expression levels of monoacylglycerol acyltransferase, diacylglycerol acyltransferase, and SCD1 mRNA in skeletal muscle.

**Fig. 6.**
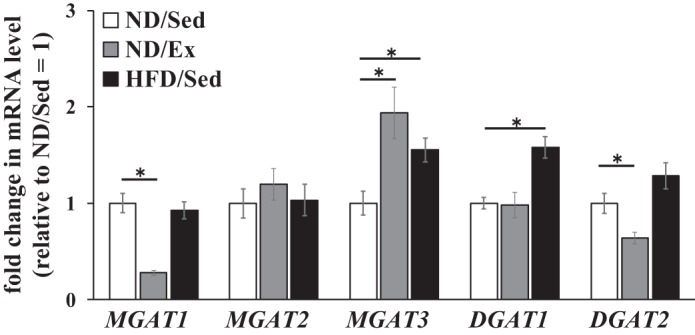
Effects of endurance exercise training and high-fat diet (HFD) on mRNA expression levels of monoacylglycerol acyltransferase (*MGAT*) and diacylglycerol acyltransferase (*DGAT*) genes in skeletal muscle. Relative mRNA levels of genes of *MGAT* and *DGAT* families in soleus muscle of normal diet (ND) plus sedentary (ND/Sed) rats, ND plus endurance exercise-training (ND/Ex) rats, and HFD plus sedentary (HFD/Sed) rats. Data are presented as means ± SE. *P < 0.05 compared with the ND/Sed rats by Dunnett’s multiple comparison test.

Monoacylglycerol acyltransferase (MGAT) catalyzes synthesis of DAG from monoacylglycerol (MAG) and is known to be expressed in skeletal muscle (19a). We found that the *MGAT1* mRNA level was markedly reduced by exercise training (Fig. 6). However, exercise training and HFD led to higher levels of *MGAT3* mRNA in soleus muscle compared with that in sedentary ND-fed rats (Fig. 6). Diacylglycerol acyltransferase (DGAT) catalyzes the synthesis of TAG from DAG. This enzyme has been shown to be expressed in skeletal muscle (6a), and we found that the *DGAT2* mRNA level was markedly reduced by exercise training (Fig. 6). In contrast, in rats that were fed HFD, soleus muscle tissue expressed higher levels of *DGAT1* mRNA than those detected in sedentary ND-fed rats (Fig. 6).

**Fig. 7.**
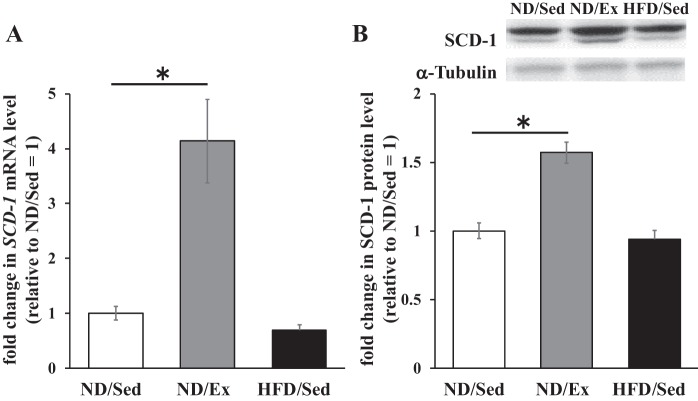
Effects of endurance exercise training and high-fat diet (HFD) on stearoyl-CoA desaturase 1 (SCD1) mRNA and protein expression in skeletal muscle. *A*: SCD1 mRNA levels in soleus muscle of normal diet (ND) plus sedentary (ND/Sed) rats, ND plus endurance exercise-training (ND/Ex) rats, and HFD plus sedentary (HFD/Sed) rats. *B*: SCD1 protein expression levels in soleus muscle of ND/Sed rats, ND/Ex rats, and HFD/Sed rats. Data are presented as means ± SE. *P < 0.05 compared with the ND/Sed rats by Dunnett’s multiple comparison test.

We next evaluated the expression of SCD1, a key enzyme for palmitoleate biosynthesis that introduces a double bond into palmitate. The mRNA and protein levels of SCD1 in soleus muscle were significantly higher in rats that had exercise training than in untrained sedentary rats on ND (Fig. 7, *A* and* B*). In contrast, the *SCD1* mRNA level was lower in rats on HFD, whereas SCD1 protein expression levels were similar in sedentary rats that received ND and HFD (Fig. 7, *A* and* B*).

## DISCUSSION

To elucidate the mechanism of the athlete’s paradox, the present study examined whether endurance exercise training and HFD feeding had distinct effects on lipid accumulation and the profile of lipid molecular species in rat skeletal muscle.

Lipid accumulation in skeletal muscle is causally related to the pathogenesis of type 2 diabetes (11, 23). Elevated DAG levels in skeletal muscle are associated with insulin resistance in obese humans and rodents (18, 34). Endurance-trained athletes also have higher DAG content in skeletal muscle than normal individuals (1). Nonetheless, endurance-trained athletes have normal insulin sensitivity. The discrepancy between consequences of lipid accumulation in endurance athletes and obese individuals is called the athlete’s paradox, and its mechanisms have not been understood.

In a good agreement with the results of a recent study (9), we observed that total DAG content in rat soleus muscle was increased by both endurance exercise training and HFD consumption (Fig. 1*A*). DAG is synthesized from MAG by esterification with a fatty acid. MGAT is the rate-limiting enzyme for MAG esterification (29), and it has been recently reported that HFD increases *MGAT3* mRNA levels in the liver (14). In the present study, we also observed that HFD consumption was associated with increased *MGAT3* mRNA levels in rat skeletal muscle (Fig. 6). Importantly, exercise training also increased the *MGAT3* mRNA level in skeletal muscle (Fig. 6). Thus alteration of DAG levels in skeletal muscle caused by exercise training and HFD may be mediated by the enhanced expression of MGAT3. At the same time, in contrast to changes in DAG content, we confirmed that endurance exercise training did not affect the total TAG level in skeletal muscle. The apparent difference in the effects of endurance exercise on DAG and TAG accumulation is likely explained by differential modulation of respective synthesis pathways. TAG is synthesized from DAG, and this reaction is regulated by the specific rate-limiting enzymes DGATs. Notably, Zhang et al. (35) reported that HFD consumption increased DGAT protein levels in the myocardium. In this study, we found that HFD consumption was associated with a higher *DGAT1* mRNA level in rat skeletal muscle (Fig. 6). Although a previous study showed that acute endurance exercise increased DGAT1 activity and protein content (27), exercise training did not affect DAGT1 expression in human skeletal muscle (2). In the present study, we also observed that endurance exercise training did not affect *DGAT1* mRNA level in skeletal muscle. Importantly, exercise training reduced the *DGAT2* mRNA level in rat skeletal muscle (Fig. 6). Thus these results suggest that the alteration of the expression level of lipid metabolic enzymes after endurance exercise modulates DAG catabolism and anabolism and may be of central importance in mediating endurance exercise training-induced DAG accumulation in skeletal muscle.

In contrast to our results, some previous studies showed that the DAG content in skeletal muscle was not affected by endurance exercise training (10, 21). Discrepancies in the outcomes of those studies and our experiments might be due to the differences in the intensity, workout time, and duration of exercise training. In our study, exercise training was with higher intensity and longer workout time compared with those in other reports. In the present study, the PGC-1 protein level in soleus muscle increased 1.7-fold after exercise training. The extent of PGC-1 expression change in our experiments was similar to that observed in a previous study that reported an increase in skeletal muscle DAG levels after exercise training (9). These results indicate a possibility that exercise intensity above a certain threshold is necessary to observe higher DAG levels in skeletal muscle. Future studies will be necessary to establish more precisely the intensity level and workout time of exercise training necessary for reliable induction of lipid accumulation and alterations of lipid metabolism enzyme levels.

Recent findings showed that specific DAG molecular species may be associated with muscle insulin resistance (4). It is well established that saturated fatty acids, such as palmitate and stearate, affect insulin sensitivity of skeletal muscle (16). In fact, palmitate was shown to impair insulin-stimulated glucose uptake in skeletal muscle cells (10, 21). Moreover, Cao et al. (6) reported that infusion of TAG containing palmitate impaired insulin-signaling pathway in mouse skeletal muscle. Those results indicate that accumulation of palmitate-containing lipids in skeletal muscle may induce muscle insulin resistance. In contrast to the effects of palmitate, monounsaturated fatty acids, such as palmitoleate, were found to enhance insulin sensitivity. It was recently shown that oral administration of palmitoleate decreased plasma glucose levels in diabetic mice (31, 36). Several pieces of evidence indicated that the effect of palmitoleate on skeletal muscle was the key factor for the glucose-lowering effect. In fact, Dimopoulos et al. (8) reported that palmitoleate increased glucose uptake via the translocation of glucose transporter type 4 in skeletal muscle cells. In addition, infusion of TAG containing palmitoleate has been shown to stimulate glucose uptake and activate the insulin-signaling pathway in mouse skeletal muscle (6). Those findings indicate that palmitate and palmitoleate exert distinct effects on insulin action in skeletal muscle and suggest that accumulation of palmitoleate-containing lipids in skeletal muscle improved muscle insulin sensitivity. Therefore, it is important to examine the effects of HFD consumption and endurance exercise training on DAG molecular species in relation to insulin resistance.

Although in our study lipid accumulation in skeletal muscle was observed both in obese rats and trained rats that received ND, we hypothesized that lipid fatty acid composition could be different in these two groups. Interestingly, the variation pattern of DAG molecular species in soleus muscle of rats that received HFD was different from that of endurance-trained rats on ND. In particular, compared with their levels in untrained, sedentary rats fed ND, levels of DAG molecular species containing C16:1 were higher in rats that underwent endurance exercise training but lower in HFD-fed rats. We found that in addition to changes in DAG species, HFD consumption altered TAG molecular species. For example, HFD-fed rats had lower levels of TAG molecular species containing C16:1 and higher levels of species containing C16:0 compared with those in sedentary ND-fed rats. Interestingly, those changes were unique to obese, HFD-fed rats and were not observed in rats that underwent endurance exercise training. Taken together, our data reveal that exercise training and HFD differentially affected the ratio of palmitate- and palmitoleate-containing lipids, which may be relevant to muscle sensitivity to insulin action. Therefore, alterations of glycerolipid fatty acid composition might be an important factor underpinning the athlete’s paradox.

Notably, it has been recently demonstrated that the concentration of palmitoleate in phospholipids of rat skeletal muscle was decreased by HFD (19). In the present study, we found that levels of PE species containing C16:1 (e.g., PE 34:3 and 38:7) were higher in rats that underwent endurance exercise training but lower in animals fed an HFD (Fig. 3). Moreover, we observed that contents of multiple PE and PC molecular species containing C16:1 in skeletal muscle in HFD-fed rats were lower than in untrained ND-fed animals. These results suggest that the fatty acid composition of phospholipids was also affected differentially by HFD and exercise training. These differences may also partly underlie dissimilar reactions of skeletal muscle to insulin action in these two settings.

A limitation of this study was that it did not reveal whether exercise and HFD caused different changes in muscle insulin action as in the case of fatty acid composition in skeletal muscles. Importantly, it has been previously reported that 8 wk of HFD intake decreased whole body glucose tolerance in rats (25, 30). Contrastingly, it was also reported that 8 wk of high-intensity endurance training increased whole body glucose tolerance in rats (13, 20). This evidence indicates that exercise and HFD exert distinct effects on glucose tolerance. Future studies should investigate these different changes in muscle insulin action thoroughly.

The mechanisms of alterations in fatty acid composition by endurance exercise training or HFD consumption have so far remained an unresolved issue. It is known that fatty acid composition of glycerolipids and phospholipids is regulated by rate-limiting enzymes controlling fatty acid metabolism. Specifically, SCD1 converts saturated fatty acids into monounsaturated fatty acids [e.g., stearate (C18:0) into oleate (C18:1) and palmitate (C16:0) into palmitoleate (C16:1)]. Importantly, SCD1 is expressed in skeletal muscle where it appears to influence fatty acid composition. Rogowski et al. (26) reported that mice overexpressing SCD1 in muscle tissue exhibited increased C16:1/C16:0 glycerolipid ratio in skeletal muscle compared with its value in wild-type obese mice. Furthermore, SCD1-overexpressing mice also showed increased glucose uptake by the muscles after insulin treatment compared with that of wild-type mice (26). It has been shown previously that SCD1 expression by skeletal muscle is influenced by chronic exercise. In fact, SCD1 protein levels in skeletal muscle were higher in endurance-trained athletes than in sedentary individuals (1). Moreover, SCD1 protein levels are upregulated in skeletal muscle after endurance exercise training (9, 17). In addition to chronic exercise, acute endurance exercise has been shown to increase SCD1 protein levels in human skeletal muscle (27). Importantly, in contrast to the effect of endurance exercise training, consumption of HFD led to lower *SCD1* mRNA levels in skeletal muscle (2). Consistent with the results of that report, we observed that the *SCD1* mRNA level in skeletal muscle was lower in rats that received HFD. Furthermore, we found that exercise training increased both mRNA and protein levels of SCD1. Collectively, our findings indicate that SCD1 is a key enzyme that mediates effects of endurance exercise training and HFD consumption on fatty acid composition in skeletal muscle.

In summary, we have demonstrated that fatty acid composition of glycerolipids and phospholipids is differentially affected by endurance exercise training and HFD consumption. Our results provide evidence that different levels of insulin sensitivity in skeletal muscle of athletes and obese individuals on the background of similar levels of accumulated lipids may be associated with quantitative alterations of fatty acid profiles in these two groups.

## GRANTS

This study was supported by Ministry of Education, Culture, Sports, Science and Technology (MEXT)/Japan Society for the Promotion of Science KAKENHI Grants 15H0597, 15H05904, 15H04708, 15H06600, 16H03205, 16K08316, 16K12940, 16K08596, 15KK0320, and 26750303 and grants from the Naito Foundation, Ono Medical Research Foundation, Uehara Memorial Foundation, Mitsubishi Foundation, Nakatomi Foundation, and Takeda Science Foundation. This study was also supported in part by Grant-in-Aid S1311011 and S1411008 from the Foundation of Strategic Research Projects in Private Universities from the MEXT and a grant of the Institute for Environmental and Gender-Specific Medicine.

## DISCLOSURES

No conflicts of interest, financial or otherwise, are declared by the authors.

## AUTHOR CONTRIBUTIONS

N.K. and S.M. conceived and designed research; N.K., K.T., H.-C.L., and D.N. performed experiments; N.K., K.T., H.-C.L., and D.N. analyzed data; N.K., K.T., H.-C.L., T.O., T.Y., and S.M. interpreted results of experiments; N.K. prepared figures; N.K. drafted manuscript; N.K., T.O., and S.M. edited and revised manuscript; N.K., K.T., H.-C.L., D.N., T.O., T.Y., and S.M. approved final version of manuscript.
